# Stromal TRIM28-associated signaling pathway modulation within the colorectal cancer microenvironment

**DOI:** 10.1186/s12967-018-1465-z

**Published:** 2018-04-10

**Authors:** Seán Fitzgerald, Virginia Espina, Lance Liotta, Katherine M. Sheehan, Anthony O’Grady, Robert Cummins, Richard O’Kennedy, Elaine W. Kay, Gregor S. Kijanka

**Affiliations:** 1 Biomedical Diagnostics Institute, Dublin City University, Dublin 9, Ireland; 2 School of Biotechnology, Dublin City University, Dublin 9, Ireland; 3 Center for Applied Proteomics and Molecular Medicine, George Mason University, Manassas, VA 20110 USA; 4 Department of Pathology, Royal College of Surgeons in Ireland and Beaumont Hospital, Dublin 9, Ireland; 5 Research Complex, Hamid Bin Khalifa University, Education City, Doha, Qatar; 6 Translational Research Institute, Immune Profiling and Cancer Group, Mater Research Institute-The University of Queensland, 37 Kent St., Woolloongabba, QLD 4102 Australia

**Keywords:** TRIM28, Colorectal cancer, Epithelium, Stroma, Prognosis, tumor antigen

## Abstract

**Background:**

Stromal gene expression patterns predict patient outcomes in colorectal cancer. TRIM28 is a transcriptional co-repressor that regulates an abundance of genes through the KRAB domain family of transcription factors. We have previously shown that stromal expression of TRIM28 is a marker of disease relapse and poor survival in colorectal cancer. Here, we perform differential epithelium-stroma proteomic network analyses to characterize signaling pathways associated with TRIM28 within the tumor microenvironment.

**Methods:**

Reverse phase protein arrays were generated from laser capture micro-dissected carcinoma and stromal cells from fresh frozen colorectal cancer tissues. Phosphorylation and total protein levels were measured for 30 cancer-related signaling pathway endpoints. Strength and direction of associations between signaling endpoints were identified using Spearman’s rank-order correlation analysis and compared to TRIM28 levels. Expression status of TRIM28 in tumor epithelium and stromal fibroblasts was assessed using IHC in formalin fixed tissue and the epithelium to stroma protein expression ratio method.

**Results:**

We found distinct proteomic networks in the epithelial and stromal compartments which were linked to expression levels of TRIM28. Low levels of TRIM28 in tumor stroma (high epithelium: stroma ratio) were found in 10 out of 19 cases. Upon proteomic network analyses, these stromal high ratio cases revealed moderate signaling pathway similarity exemplified by 76 significant Spearman correlations (ρ ≥ 0.75, p ≤ 0.01). Furthermore, low levels of stromal TRIM28 correlated with elevated MDM2 levels in tumor epithelium (p = 0.01) and COX-2 levels in tumor stroma (p = 0.002). Low TRIM28 epithelium to stroma ratios were associated with elevated levels of caspases 3 and 7 in stroma (p = 0.041 and p = 0.036) and an increased signaling pathway similarity in stromal cells with 81 significant Spearman correlations (ρ ≥ 0.75, p ≤ 0.01).

**Conclusions:**

By dissecting TRIM28-associated pathways in stromal fibroblasts and epithelial tumor cells, we performed comprehensive proteomic analyses of molecular networks within the tumor microenvironment. We found modulation of several signaling pathways associated with TRIM28, which may be attributed to the pleiotropic properties of TRIM28 through its translational suppression of the family of KRAB domain transcription factors in tumor stromal compartments.

**Electronic supplementary material:**

The online version of this article (10.1186/s12967-018-1465-z) contains supplementary material, which is available to authorized users.

## Background

The crosstalk between epithelial cells and the non-epithelial cellular component of tumor stroma exerts substantial influence on the severity and aggressiveness of cancer [1]. Stromal fibroblasts, in particular, contribute to the crosstalk through secretion of cytokines and growth factors thereby triggering signaling pathways in tumor cells [2, 3]. In colorectal cancer, tumor stroma is known to impact on patient outcomes [4], with stromal genetic instability being a key contributing factor [5]. Recent transcriptomic profiling studies confirmed these earlier findings and have defined extensive stromal gene expression profiles linked to poor prognosis and to the induction of epithelial–mesenchymal transition (EMT) through TGF-ß signaling in colorectal cancer [6]. Since EMT is associated with distinct gene profiles in colorectal cancer patients with poor outcomes [7] and is thought to be regulated by a wide range of transcription factors [8], the impact of the stromal compartment on the tumor microenvironment might be determined by transcriptional control of a wide range of genes in stromal fibroblasts.

Tripartite motif-containing 28 (TRIM28) is a nuclear corepressor involved in transcriptional regulation of a large number of transcription factors belonging to the Krüppel-associated box (KRAB) repressor domain family of zinc finger proteins [9, 10]. TRIM28 has been recently shown to exhibit transcriptional control over a plethora of regulatory networks and cell re-programming pathways [11–13]. TRIM28 is an essential component of the fibroblast transcription site-1 activator transcription complex required for induction of EMT [14] and has been shown in cancer to further contribute to EMT via regulation of E- and N-cadherins [15]. In addition, TRIM28 can mediate communication between cells through regulation of senescence in fibroblasts causing the induction of secretory phenotype which includes the secretion of interleukins and other pro-inflammatory molecules [16].

We have previously shown that TRIM28 is overexpressed in colorectal cancer [17] and that its stromal expression is an independent marker of disease relapse and patient survival [18]. More recently, tumor promoting effects of TRIM28 were observed in glioma and breast cancer [19, 20]. In this paper, we sought to further investigate how differences in expression patterns of TRIM28 in epithelial and stromal tissue compartments in colorectal cancer can influence patient survival. Since regulatory mechanisms of the corepressor TRIM28 are likely to be imposed on pan-transcriptional level through co-repression or co-activation of over 700 distinct KRAB domain transcription factors [10, 21], in this study we take a proteomic systems approach which allows us to investigate signaling pathway activation differentially in tumor epithelium and stromal compartments. We here employ laser capture microdissection to isolate enriched populations of epithelial and stromal cells and perform a reverse phase protein array-based proteomic analysis of selected cancer signaling endpoints to investigate the underlying molecular interactions at play in relation to TRIM28 within the tumor microenvironment.

## Methods

### Patient cohort and tissue specimens

The study was approved by Ethics (Medical) Research Committee at Beaumont Hospital, Dublin, Ireland. Informed consent was obtained from all patients. Patients with a history of cancer and neo-adjuvant treatment were excluded. A total of 19 cases with late-stage (stages III and IV) colorectal cancer (CRC) were investigated. All patients were diagnosed with CRC between 2012 and 2013. The median age of the patients at the time of diagnosis was 67 (range 47–88 years) with 10 male and 9 female patients. In total, 15 patients had colonic carcinoma, whilst 4 had rectal carcinomas. Clinical and pathological parameters of the patient cohort are shown in Table 1.

**Table 1:** Clinicopathological details of patient cohorts

Factor	Number of patients (*n *= 19)	%
Gender		
Female	9	47.4
Male	10	52.6
Age (years)		
Median	67	–
Range	47–88	–
< 65	8	42.1
≥ 65	11	57.9
Tumor site		
Colon	15	78.9
Rectum	4	21.1
Tumor stage^a^		
T3	13	68.4
T4	6	31.6
Node stage^a^		
N0	11	57.9
N1	2	10.5
N2	6	31.6
Metastasis stage^a^		
M0	17	89.5
M1	2	10.5
Lymphovascular invasion		
Yes	7	36.8
No	12	63.2
Differentiation		
Well	0	0
Moderately	17	89.5
Poorly	2	10.5

*n* number of patients, *T* tumor, *N* node, *M* metastasis

^a^TNM were staged according to the 5th edition of the AJCC Cancer Staging Manual

Both, fresh-frozen and formalin-fixed and paraffin embedded (FFPE) tissue samples were obtained for each case. FFPE tissues were used for immunohistochemical analysis, while fresh-frozen tissues were used for laser capture microdissection (LCM) and reverse phase protein array (RPPA) analysis. A pathologist identified and collected areas of invasive carcinoma from the tumor mass for fresh-frozen preservation and FFPE. Local resection and standard fixation protocols were carried out in all cases. Each FFPE block was sectioned and stained with hematoxylin and eosin (H&E) and graded by a consultant pathologist to confirm pathological stage and grade of the tumors. All fresh tissue samples for RPPA analysis were snap-frozen in liquid nitrogen and processed uniformly and rapidly to ensure preservation of molecular endpoints. The time from removal of a colectomy specimen to snap-freezing of samples was < 20 mins. Fresh-frozen tissue samples were stored at − 80 °C.

### Immunohistochemistry staining and assessment

Immunohistochemistry (IHC) and scoring were carried out using an anti-TRIM28 rabbit monoclonal antibody (mAb) (C42G12, Cell Signaling Technology Inc., Danvers, MA, USA), as previously described [18]. The degree of nuclear TRIM28 staining was evaluated for epithelial and stromal tissue separately and scored as follows: absence of staining (score = 0), weak staining (score = 1+), moderate staining (score = 2+) and strong staining (score = 3+). TRIM28 epithelium to stroma ratios were computed based on a Protein Expression Ratio method using IHC scores as previously described [18]. Briefly, high TRIM28 Expression Ratio was defined as at least 2 units of difference in staining intensity (e.g. epithelium strong [3+] and stroma weak [1+], or epithelium moderate [2+] and stroma negative [0]). Low TRIM28 Expression Ratio was defined as 1 or 0 units of difference in staining intensity (e.g. epithelium moderate [2+] and stroma weak [1+], or epithelium weak [1+] and stroma weak [1+]). A previous study in our lab has shown that the inter-observer variability of IHC scoring is as low as 7% [22]. In cases where there were discrepancies between the scorers, a consensus was reached after a joint review using a multi-headed microscope.

### Laser capture microdissection and reverse phase protein arrays

Laser capture microdissection (LCM) was performed to isolate separate populations of epithelial and stromal cells for cell signaling analysis as described previously [23–25]. Briefly, consecutive 8 µm thick frozen tissue sections were cut using a cryostat for each sample and mounted on glass slides. Then, using an infrared-based laser capture system (ArcturusXT, Applied Biosystems, San Francisco, CA, USA), approximately 20,000 epithelial and stromal cells were removed for each frozen tissue sample. To account for possible heterogeneity within the tissue sample, multiple separate areas within each patient sample were micro dissected and no attempt was made to target specific regions within the tumor. Tissue processing and preparation of tissue lysates have been described previously [26, 27].

Reverse phase protein arrays (RPPA) were generated as previously described [25, 28, 29]. Each array contained epithelial and stromal cell lysates for all 19 cases and each lysate was printed in a 2-fold dilution curve representing undiluted lysate, 1:2, 1:4 and 1:8 dilutions. Control lysates were printed in a twofold dilution curve. All RPPAs were incubated for 2 h at 80 °C to allow fixation and then stored with desiccant at − 20 °C. Quality control samples were printed on the RPPA to ensure protein deposition and immunostaining reactivity [30]. These included A431 cell lines (± EGF stimulation; BD Pharmingen, San Diego, CA, USA) and a pooled sample of CRC cases.

### RPPA immunostaining, image acquisition and data analysis

RPPA slides were blocked (I-Block, Applied Biosystems) for 2 h prior to immunostaining. Immunostaining was conducted on a Dako Autostainer (Catalyzed Signal Amplification (CSA) kit, Dako). Each slide was incubated with a single primary antibody at room temperature for 30 min. The negative control slide was incubated with antibody diluent (Dako) instead of a primary antibody. For normalization purposes, a slide was incubated with anti-ssDNA antibody (1:15,000; IBL International GmbH). The secondary antibodies were goat anti-rabbit (1:10,000; Vector Laboratories), or rabbit anti-mouse IgG (1:10; Dako) depending on the host species of the primary antibody. Amplification was achieved using horseradish peroxidase-mediated biotinyl tyramide with chromogenic detection (diaminobenzidine; Dako). In order to determine the total protein concentration in each sample, two RPPA slides were stained with Sypro Ruby Protein Blot Stain (Molecular Probes, Eugene, OR, USA) and visualized with NovaRay Image Acquisition Software (Alpha Innotech, San Leandro, CA, USA).

In total, 30 primary antibodies specific to known signaling endpoints were used to measure phosphorylation and protein levels using RPPAs (see Additional file 1). In an effort to elucidate the underlying proteomic mechanisms responsible for the previously reported significantly different 5-year survival between TRIM28 high and low ratio patients [18], many endpoints related to known catabolic pathways essential for organismal homeostasis such as autophagy and apoptosis were chosen. Several endpoints that are known modulators of TRIM28 function were also included.

RPPA slides were scanned on a flatbed scanner (UMAX 2100XL; white balance 255, black 0, middle tone 1.37, 600 dpi, 14 bit) and spot intensity was analyzed using the Image Quant v5.2 software package (Molecular Dynamics). To normalize data, the relative intensity for each protein spot was divided by the ssDNA relative intensity for the corresponding spot [31] and data reduction was carried out using a VBA Excel macro, RPPA Analysis Suite [32]. One case did not meet the quality criteria as the spot intensities were not significantly different from the negative control and therefore was excluded from proteomic network analysis. All staining intensities are listed in Additional file 2: Table S2.

### Proteomic network analysis

Proteomic network analysis was performed as previously shown [25, 33]. Spearman ρ correlation analysis with ρ ≥ 0.75 and *p *≤ 0.01 was used to build proteomic network graphs (Gephi 0.9, The Gephi Consortium, Paris, France, http://www.gephi.org). Proteomic networks were drafted based on 18 CRC cases (one excluded) and classified into two groups based on their epithelial to stromal TRIM28 expression ratio score; TRIM28 low ratio group (n = 9) representing good patient prognosis and TRIM28 high ratio group (n = 9) representing poor patient prognosis [18]. In order to identify the differences in the proteomic signature of the cohorts, significant correlations that were common in both TRIM28 ratio groups in the same tissue compartment (epithelial or stromal) were excluded and only exclusive significant correlations were used to construct the proteomic network graphs. For example, correlations in the epithelial compartment that were common in both TRIM28 high and low ratio cohorts, were excluded and only correlations that were unique to the TRIM28 high or low ratio groups were used to construct the proteomic network graphs for each group.

Nodes in the network represent proteins quantified by RPPA; the bigger the node, the more significant correlations relative to that protein. Each line connecting 2 nodes represents a significant correlation between the nodes; the thicker the line, the higher the Spearman ρ correlation between those two proteins. Proteins within the network are grouped into various subgroups on the basis of Spearman ρ values and the number of connections among a group of nodes. Strongly correlated nodes are represented close to each other and with the same color, with each color representing a different subgroup. The number of subgroups within each proteomic network is determined by the software.

### Statistical analysis

All tests were analyzed using SPSS 21.0 software (SPSS, Chicago, IL, USA) and JMP 5.1.2 (SAS Institute Inc., Cary, NC, USA). The Spearman rank correlation coefficient, ρ, was calculated for each protein pair in the RPPA cohort; ρ ≥ 0.75 with *p* ≤ 0.01 was considered significant. An independent-samples *t* test was used to compare the expression levels of each endpoint between groups, the findings were considered statistically significant at p < 0.05.

## Results

### TRIM28 expression in tumor epithelium and stroma

**Fig. 1:**
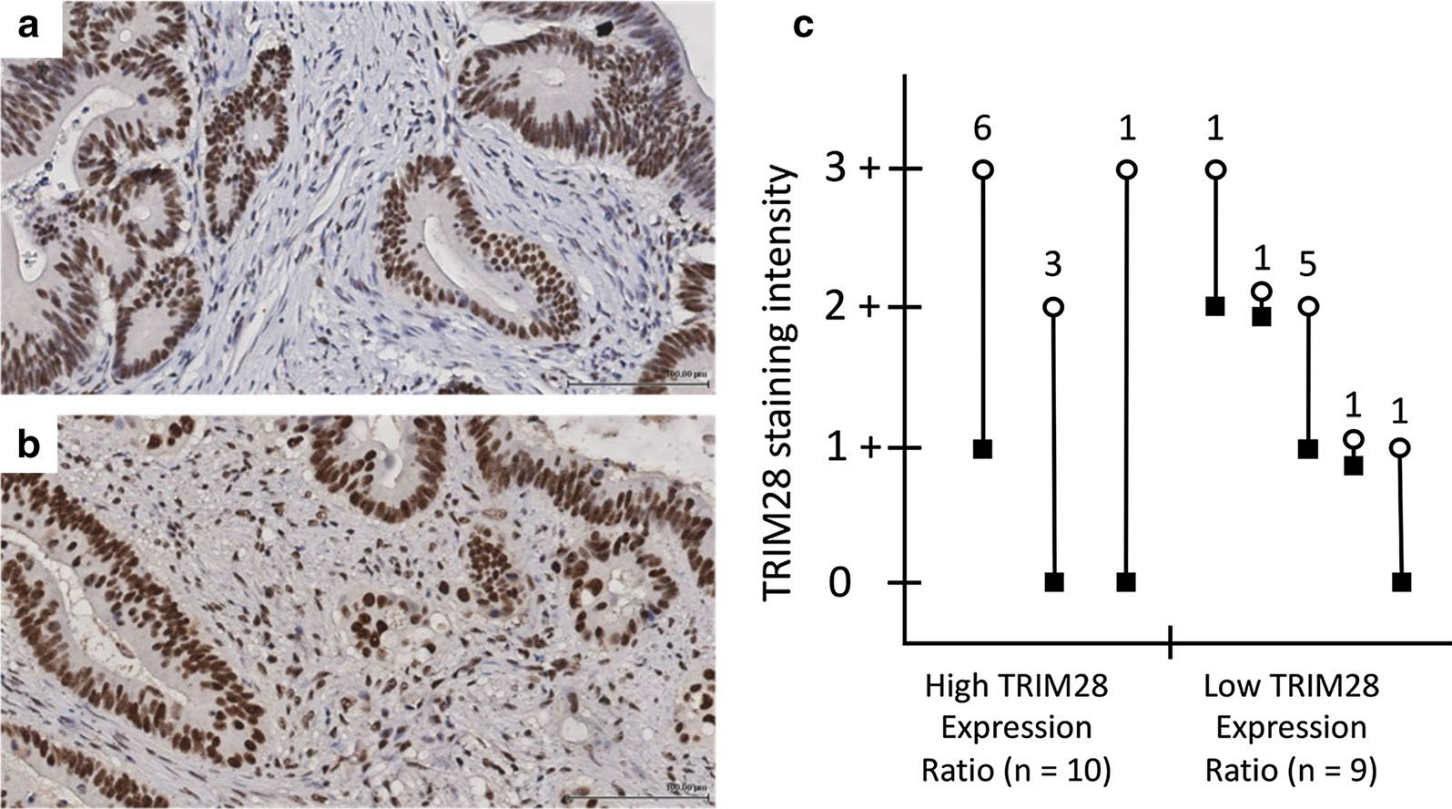
Epithelial to stromal TRIM28 Expression Ratios in colorectal cancer. **a** Moderate (2+) expression of TRIM28 in epithelial cells and absence (0) of staining in stromal compartments resulting in a high epithelial to stromal TRIM28 Expression Ratio score (×200). **b** Moderate (2+) expression of TRIM28 in both epithelial and stromal cells resulting in a low epithelial to stromal TRIM28 Expression Ratio score (×200). **c** A graph of distributions of all TRIM28 Expression Ratios cases. Absence of staining (0), weak (1+), moderate (2+), or strong (3+) TRIM28 staining intensities were found in both epithelial and stromal tissue compartments. The TRIM28 Expression Ratios are labelled by (open circle) epithelial and (filled square) stromal symbols, which are linked with straight lines for matched cases. The numbers above the connecting lines depict the total number of cases for each specific distribution of epithelial to stromal TRIM28 Expression Ratios. All tissues were stained using IHC with an anti-TRIM28 C42G12 antibody

We have previously shown that the assessment of TRIM28 expression levels, when it encompasses epithelial and stromal tissue compartments, can serve as an independent marker of relapse and patient survival in colorectal cancer [34]. In order to assess stromal and epithelial TRIM28 levels in this cohort, we separately scored TRIM28 in epithelial carcinoma cells and stromal fibroblasts in 19 FFPE tissues using IHC. Strong epithelial staining (3+) for TRIM28 was found in 8 cases, moderate staining (2+) in 9 and weak staining (1+) in 2. Cells in the stromal compartment showed moderate TRIM28 staining (2+) in 2 cases, weak staining (1+) in 12 cases and absence of staining (0) in 5 cases (Fig. 1).

The tissue-compartment protein expression ratio method developed for reciprocal tumor microenvironment assessment [34], allowed us to divide the 19-case CRC cohort in 2 groups; the TRIM28 high epithelium: stroma ratio group, which is mainly characterized by low levels of TRIM28 in tumor stroma (Fig. 1a) and the low epithelium: stroma ratio group, which shows more similar TRIM28 expression levels in both, epithelial and stromal compartments (Fig. 1b). Additional examples for epithelial to stromal expression variations and their assigned ratios are shown (Additional file 3). Using the IHC scoring data, the ratio method resulted in 10 cases being assigned to the TRIM28 high ratio group and 9 cases to the low ratio group (Fig. 1c). In the high ratio group, one case had high epithelial TRIM28 levels and absence of TRIM28 in stroma expression, 6 cases had high levels of epithelial TRIM28 and weak staining in the stroma and 3 cases had moderate levels of TRIM28 in the tumor epithelium with an absence of staining for TRIM28 in stroma (Fig. 1c). Most cases in the TRIM28 low ratio group had weak to moderate expression in both, epithelium and stroma. One case had strong epithelial expression and another case presented with absence of stromal TRIM28 staining. The distribution of IHC scores allotted to both TRIM28 expression ratio groups is shown in Fig. 1c.

### High ratio TRIM28 stromal networks are linked to tumor progression

We next sought to understand the underlying molecular interplay associated with TRIM28 expression ratios and signaling pathways linked to cancer. RPPAs were immunostained against 30 signaling endpoints and the resulting quantitative data were used for proteomic network analyses in 9 TRIM28 high ratio and 9 TRIM28 low ratio cases (see Additional file 2). The Spearman’s rank-order correlation analysis revealed that 55 of 435 possible protein pair correlations were shared (ρ ≥ 0.75, *p* ≤ 0.01) between stroma of TRIM28 high and low ratio cases (see Additional file 4).

**Fig. 2:**
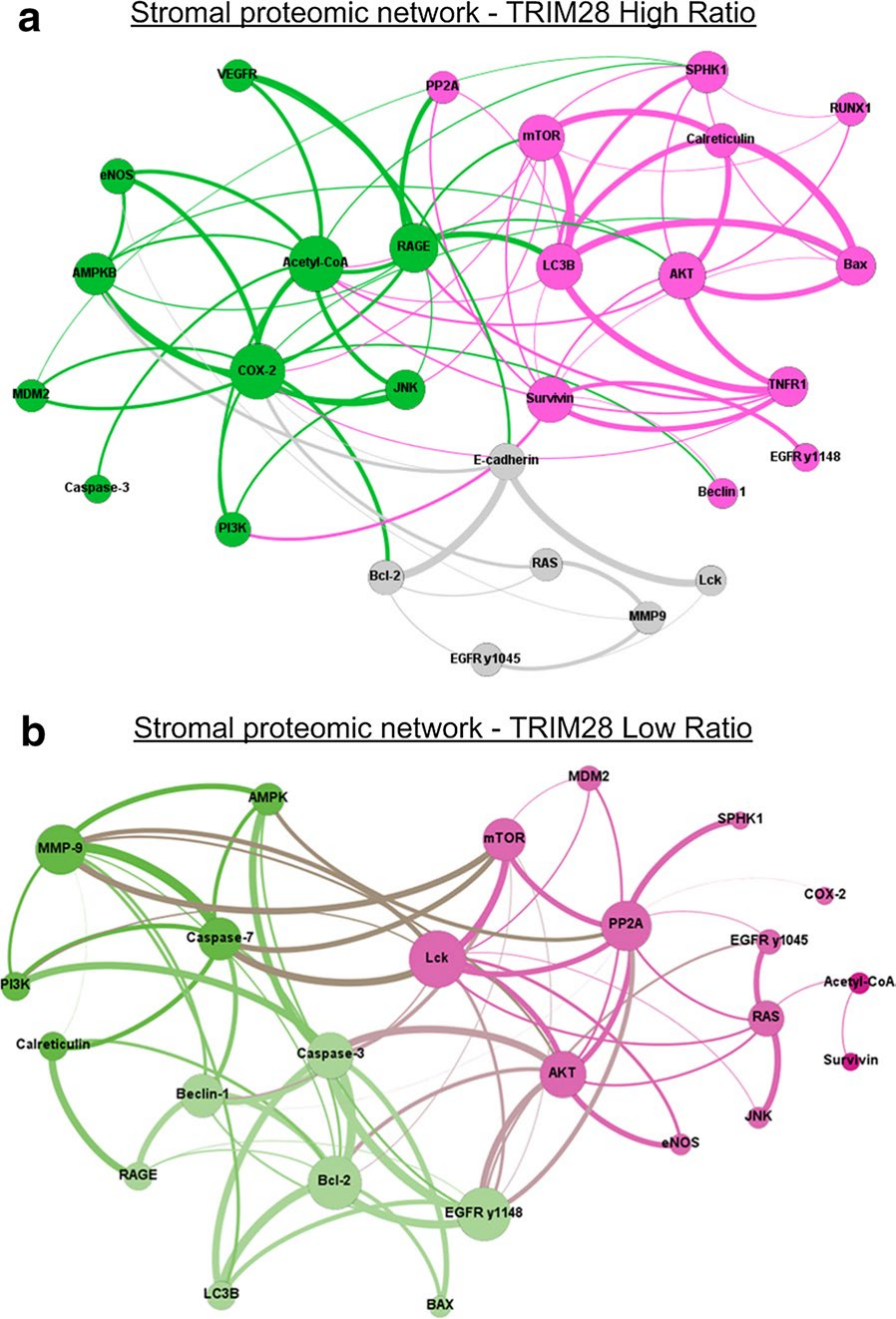
Stromal proteomic networks for TRIM28 high (**a**) and low (**b**) ratio cases. Both, TRIM28 high (**a**) and TRIM28 low ratio (**b**) stromal tissue proteomic networks consist of three main sub-networks (green, pink and light green). Dominant nodes in the high ratio sub-networks are proteins linked to tumor progression and nodes prominent in the low ratio sub-networks are proteins linked to apoptosis. Nodes represent proteins quantified by RPPA; the bigger the node, the more significant correlations relative to that protein. Each line connecting 2 nodes represents a significant correlation between the nodes; the thicker the line, the higher the Spearman ρ correlation. Proteins are grouped on the basis of Spearman ρ values and the number of connections among a group of nodes; strongly correlated nodes are represented close to each other and with the same color

In addition, the high ratio TRIM28 stromal proteomic network revealed a moderate number of 76 exclusive signaling endpoints associations, which were not present in TRIM28 low ratio stoma (see Additional file 4). As shown in Fig. 2a, the stromal high ratio network is divided into three sub-networks. The proteomic network is dominated by nodes linked to tumor progression, including acetyl-CoA, LC3B, COX-2 and Survivin as prominent endpoints.

### Low ratio TRIM28 stromal networks are linked to apoptosis

The low ratio TRIM28 stromal network showed an overall higher number of exclusive signaling endpoints with 81 significant associations when compared to high ratio TRIM28 stoma (see Additional file 4). As shown in Fig. 2b, the network is also divided in three sub-groups, these however are considerably different to the high ratio networks and are dominated by endpoints linked to apoptosis, such as the activated caspases 3 and 7 and PP2A. The association with apoptotic proteins is not seen in the TRIM28 high ratio stromal network and shows that the proteomic architecture of the stromal tissue compartment is significantly different in TRIM28 high and low ratio patients and hence may account for differences in patient outcomes seen in both colorectal cancer groups.

### High ratio TRIM28 epithelial networks are associated with cellular pro-survival pathways

In order to obtain a complete picture of potential molecular tumor microenvironment characteristics, we performed an equivalent Spearman rank correlation mapping for the epithelial compartments of the patient cohort. The 30-endpoint Spearman rank analysis revealed that 87 of 435 possible protein pair correlations were shared between epithelia of TRIM28 high and low ratio cases (see Additional file 5), indicating more similar signaling patterns between both groups when compared to the stroma counterpart.

**Fig. 3:**
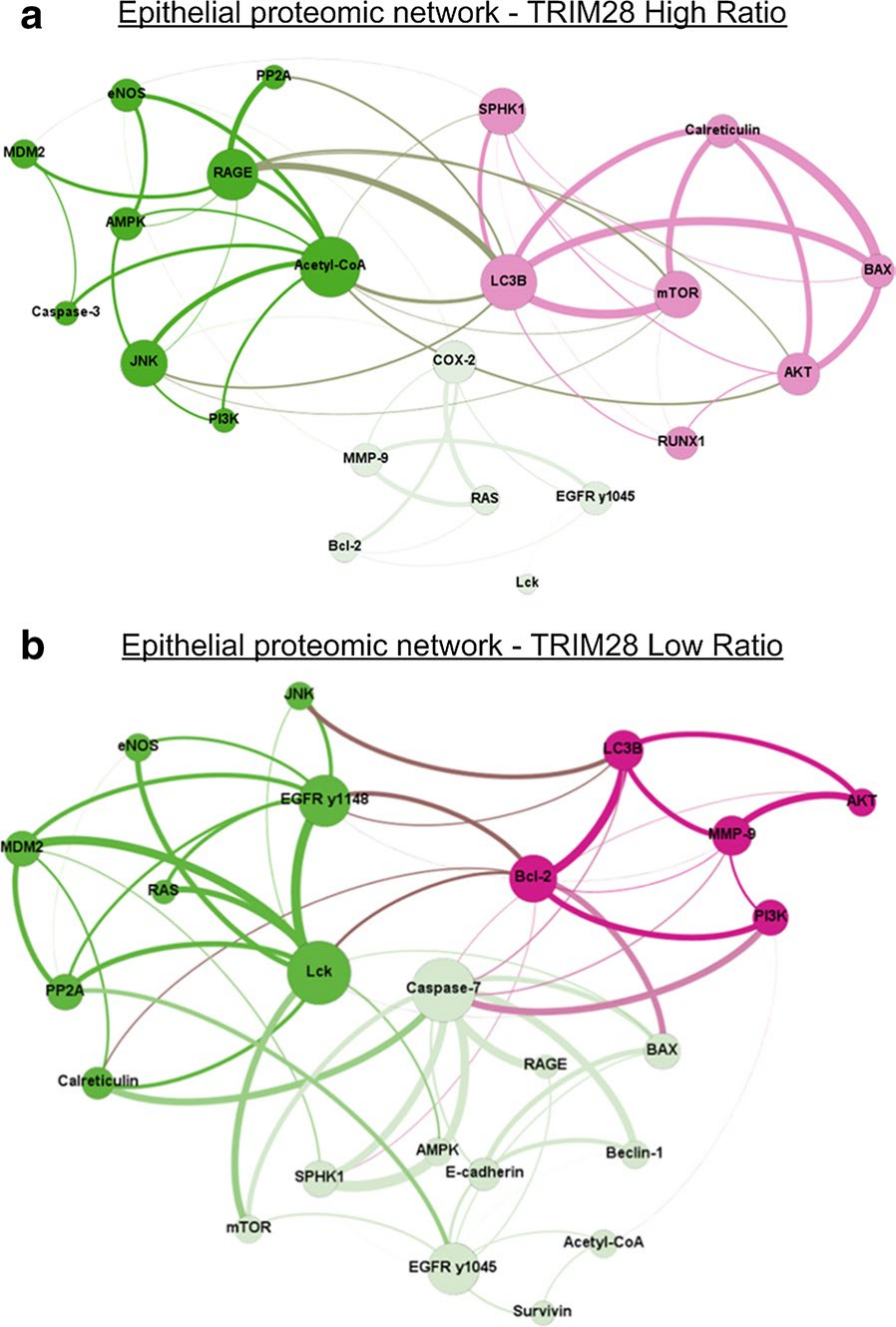
Epithelial proteomic networks for TRIM28 high (**a**) and low (**b**) ratio cases. Both, TRIM28 high (**a**) and TRIM28 low ratio (**b**) epithelial tumor tissue proteomic networks consist of three main sub-networks (green, pink and light green). Dominant nodes in the high ratio networks represent pro-survival pathways. Prominent nodes in the low ratio proteomic networks represent pro-apoptotic pathways. Nodes represent proteins quantified by RPPA; the bigger the node, the more significant correlations relative to that molecule. Each line connecting 2 nodes represents a significant correlation between the nodes; the thicker the line, the higher the Spearman ρ correlation. Proteins are grouped on the basis of Spearman ρ values and the number of connections among a group of nodes; strongly correlated nodes are represented close to each other and with the same color

Fifty-five protein pairs correlated (ρ ≥ 0.75, *p* ≤ 0.01) exclusively in the epithelium of TRIM28 high ratio cases and were entirely absent in TRIM28 low ratio cases (see Additional file 5). Detailed graphical representation of those high ratio-exclusive associations of protein expression and phosphorylation in epithelial compartments is shown in Fig. 3a. The TRIM28 high ratio epithelial proteomic network is associated with several pro-survival endpoints including cell–cell adhesion (E-cadherin) and autophagy (AKT, Survivin).

### Low ratio TRIM28 epithelial networks are associated with pro-apoptotic signaling

The proteomic network analysis of tumor epithelium in the TRIM28 low ratio group revealed statistically significant protein pair correlations in 69 signaling endpoints (ρ ≥ 0.75, *p* ≤ 0.01) (see Additional file 5). Those pairs were exclusive to the epithelium of TRIM28 low ratio cases and were absent in TRIM28 low ratio epithelium (Fig. 3b). The proteomic network is dominated by pro-apoptotic proteins represented, among others, by activated caspase-7 and the anti-apoptotic regulator BCL-2. Those nodes and protein correlations are noticeably diminished in the high ratio epithelial proteomic network. In addition, the proto-oncogene Lck, another prominent node in the TRIM28 low ratio epithelial network, was also identified as a dominant node in TRIM28 low ratio stromal network (Figs. 2b, 3b).

### Stromal MDM2 and COX-2 levels in high ratio TRIM28 colorectal cancer

**Fig. 4:**
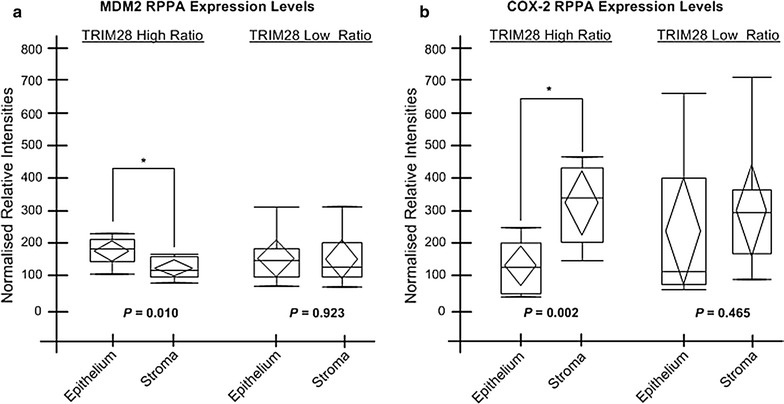
High ratio TRIM28 stromal tissue shows low levels of MDM2 and high levels of COX-2. Box-plot diagrams of **a** MDM2 and **b** COX-2 represent RPPA intensity levels in the epithelium and stroma of TRIM28 high and low ratio cases. MDM2 is downregulated in stroma of TRIM28 high Ratio cases (p = 0.010, **a**). COX2 is elevated in stroma in TRIM28 high ratio cases were found (p = 0.002, **b**). The median (line within the box), mean (center of the diamond), 25th and 75th percentiles and maximum and minimum values are all displayed on each box-plot

We next compared expression levels of 30 signaling proteins between tumor epithelial and stromal compartments in either TRIM28 high or low ratio cases. Our analysis revealed that expression levels of MDM2 are significantly lower in stroma of TRIM28 high ratio cases when compared to corresponding epithelial compartments (*p* = 0.010, Fig. 4a). No statistically significant differences in MDM2 levels were found in low ratio cases (*p* = 0.923, Fig. 4a). In contrast, stromal expression levels of COX-2 were significantly elevated in high ratio cases when compared to tumor epithelium (*p* = 0.002, Fig. 4b). There are no significant differences in COX-2 intensities between epithelium and stroma of TRIM28 low ratio cases (*p* = 0.465, Fig. 4b). These findings further substantiate a molecular disparity between TRIM28 high and low ratio cases. Our earlier findings, for instance, show that TRIM28 may interact with MDM2 to influence the levels of the tumor suppressor p53 and this may account for significantly poorer 5-year overall survival and 5-year ‘recurrence-free’ survival seen in TRIM28 high ratio colorectal cancer patients [18].

### Stromal caspases 3 and 7 levels in high ratio TRIM28 colorectal cancer

**Fig. 5:**
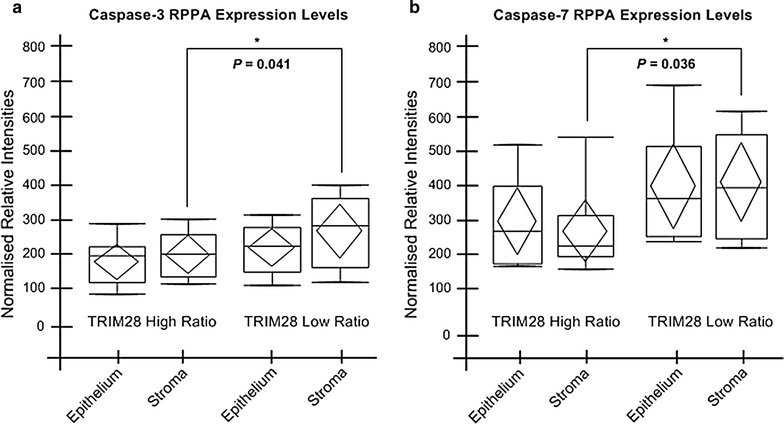
Caspase-3 and caspase-7 protein levels are lower in the stroma of TRIM28 high ratio patients. **a** Caspase-3 and **b** caspase-7 RPPA intensity levels in the epithelium and stroma of TRIM28 high and low ratio patients are represented by box-plot diagrams. There is significantly less active caspase-3 and -7 present in the stroma of TRIM28 high ratio patients than in the stroma of TRIM28 low ratio patients (p = 0.04 and p = 0.036, respectively). The median (line within the box), mean (center of the diamond), 25th and 75th percentiles and maximum and minimum values are all displayed on each box-plot

The expression levels of all investigated signaling endpoints were then analyzed across epithelial and stromal data regardless of their TRIM28 ratio scores. We found significantly less activated caspase-3 and caspase-7 expressed in the stroma of TRIM28 high ratio patients compared to stroma of low ratio patients (*p* = 0.041, Fig. 5a; *p* = 0.036, Fig. 5b). Activated caspases are the main executioners of apoptosis in the cell. Since the reduction of stromal apoptosis was previously shown to be an independent prognostic factor for poorer overall survival and disease-recurrence in CRC [35], the lower levels of activated caspases seen in the stroma may be another contributing factor to the poorer outcomes in colorectal cancer patients with TRIM28 high ratios.

## Discussion

The cancer microenvironment is a complex composite of cell types and secretory factors difficult to replicate experimentally in vitro. In this study, we have therefore taken a differential proteomic approach to investigate TRIM28-associated molecular pathway activation directly in colorectal cancer tissue compartments. TRIM28 is a marker of disease relapse and poor survival in colorectal cancer that engages different molecular mechanisms in the context of its expression within the tumor microenvironment. Since TRIM28 can regulate several hundred different genes in its role as a transcriptional co-repressor [11–13], we investigated its role within LCM-enriched stromal and epithelial compartments derived from patient tumor tissue. This study demonstrates distinctive molecular networks associated with TRIM28 expression in stromal fibroblasts and epithelial tumor cells.

The modulation of signaling pathways found in this study may be attributed the pleiotropic properties of TRIM28, specifically impacting tumor-associated stromal compartments. Altered protein expression in stromal cells has been shown in many malignancies including lung, prostate, breast cancer and colorectal cancer to be prognostic of patient survival [36–39]. We have previously shown that high TRIM28 expression ratios between stromal and epithelial compartments of colorectal carcinomas are associated with poor patient survival and disease recurrence [34]. The findings of this current study suggest that the aggressiveness of TRIM28 high ratio cancers is mediated through activation of tumor progression pathways in stromal compartments and cellular pro-survival pathways in tumor epithelium.

We found cellular pro-survival mechanisms associated with TRIM28 high ratio in the epithelial compartments, which were featured through a central involvement of cell–cell adhesion (E-cadherin) and autophagy (AKT, Survivin) pathways. Activated AKT mediates downstream responses, including cell survival, growth, proliferation, cell migration and angiogenesis by phosphorylating a range of intracellular signaling proteins. Survivin, on the other hand, is a member of the apoptosis (IAP) inhibitor family that inhibits caspase activation, thereby leading to negative regulation of apoptosis or programmed cell death. Activated caspases 3 and 7 are noticeably diminished in this protein interaction network. In contrast, the TRIM28 low ratio epithelial proteomic network is associated with pro-apoptotic proteins including activated caspase-7 and the apoptotic regulator BCL-2.

Our earlier studies propose that during the epithelial to mesenchymal transformation of tumor cells, some carcinoma cells can take on characteristics of stromal fibroblasts [40]. EMT is thought to be required physiologically during embryogenesis, but its persistence in tumor cells is suggested to play a role in the promotion of an invasive phenotype. Fibroblasts produced by EMT express a gene encoding fibroblast-specific protein 1 (FSP1), which is regulated by a promoter element called fibroblast transcription site-1 (FTS-1) [41]. TRIM28 interacts with FTS-1 resulting in the transcriptional activation of genes encoding the EMT proteome, suggesting that TRIM28 plays a key role in fibroblast formation [14]. Furthermore, epithelial cells may lose their epithelial phenotype during EMT, which often manifests through the downregulation of E-cadherin and hence the loss of specialized cell–cell contacts [42, 43]. These findings, together with the dominant role of E-cadherin in the High Ratio epithelial network suggest active EMT process undergoing in those cancers.

We demonstrate that there is significantly more epithelial than stromal MDM2 in TRIM28 High Ratio patients and this is in line with our previous findings that TRIM28 is overexpressed in epithelial cancer tissue. MDM2 plays an important role in the regulation of p53 by inhibiting its transcriptional activity, controlling its subcellular localization, and by modulating its protein stability [44]. A number of TRIM proteins modulate the abundance and the activity of p53 and/or MDM2 and, therefore, can influence p53′s tumor-suppressive activity [45]. MDM2 is a RING domain ubiquitin E3 ligase and a major regulator of the tumor suppressor p53. Importantly, TRIM28 was previously identified as an MDM2-binding protein and shown to form a complex with MDM2 and p53 in vivo [46]. TRIM28 interacts with MDM2 resulting in an enhanced deacetylation of p53 and a reduced transcriptional activity [46]. Antagonism of the MDM2-p53 interaction activates p53 signaling leading to a regression in human tumors in preclinical cancer models [47]. Our results suggest that high epithelial expression of both TRIM28 and MDM2, as seen in TRIM28 high ratio patients, results in the decrease of p53′s tumor suppressing activity.

Lck is one of the dominant nodes in both of the TRIM28 low ratio networks, and Lck overexpression has previously been linked to improved survival [48]. In recent years, cancer immunotherapy has been gaining greater significance in the treatment of cancer, in particular in adoptive cell transfer (ACT). The principle of ACT is that T-cells are genetically engineered to recognize antigens on the surface of cancer cells and destroy these cells [49]. Lck plays an essential role in the selection and maturation of developing T-cells in the thymus and in the function of mature T-cells [50]. The role of Lck in T-cell immunity may affect cancer cells in a similar manner to ACT, thereby contributing to low cancer recurrence rates seen in TRIM28 low ratio patients [18]. The Lck-related immunity may have further-reaching implications for TRIM28 as several TRIM family members, including TRIM28, have been shown to act as antigens eliciting autoantibody responses in cancer [17, 51] and autoimmune disease [52, 53].

We have previously shown that the expression levels of COX-2 is related to lymph node metastasis, advanced Dukes staging, and poorer long-term outcome for patients with colorectal cancer [54]. In this study we show that COX-2 is also overexpressed in the stroma of TRIM28 high ratio patients. COX-2 plays an important role in the inflammatory response and is generally downregulated under normal conditions in most cells, but elevated levels are found during inflammation. The overexpression of COX-2 is associated with various types of cancer and the expression levels are generally proportional to cancer aggressiveness [55, 56]. Low COX-2 expression in epithelial cells and upregulation in stroma, however, has been shown to be indicative of tumor progression in laryngeal squamous cell carcinoma [57]. The significance of COX-2 as a driver of tumorigenesis illustrates that differential proteomic approaches investigating broader downstream implications of key transcriptional modulators, as taken in this study, may reveal key microenvironmental association ultimately contributing to clinical outcomes.

## Conclusion

Taken together, our findings suggest that the proteomic architecture of the stromal tissue compartment is significantly different in TRIM28 high and low ratio patients, favoring apoptotic processes in the low ratio patients, which may be ultimately linked to better outcome seen in this patient cohort. The study highlights the importance of evaluating the expression levels of biomarkers directly in the tumor microenvironment as an alternative to other in vitro models. By dissecting the effects of TRIM28 in stromal fibroblasts and epithelial tumor cells, we were able to elucidate the complex relationship between stromal and epithelial compartments in CRC. Our approach offers insights into the tumor suppressive and tumor promoting effects of the highly pleiotropic protein TRIM28, thereby reinforcing its value as a prognostic biomarker and a potential therapeutic target.

## Additional files

**Additional file 1: Table S1.**

**Additional file 2: Table S2.**

**Additional file 3.** Additional examples of TRIM28 expression ratios.

**Additional file 4: Table S3.** Significant Spearman’s Rho correlations from RPPA measurements of stromal cells.

**Additional file 5: Table S4.** Significant Spearman’s Rho correlations from RPPA measurements of epithelial cells.
