# Supplementary figures and images for: Stromal TRIM28-associated signaling pathway modulation within the colorectal cancer microenvironment
Source: J Transl Med. 2018 Apr 10;16:89. doi: 10.1186/s12967-018-1465-z (PMC5891886; doi:10.1186/s12967-018-1465-z)

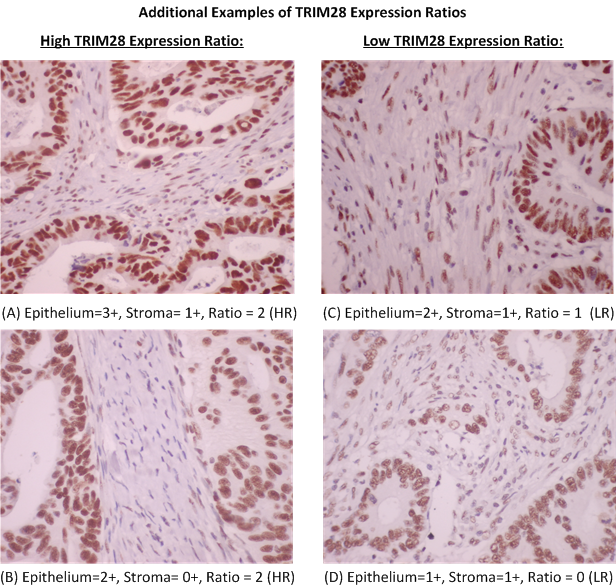

Supplement: Supplementary file 3 — Additional file 3. Additional examples of TRIM28 expression ratios. [file 12967_2018_1465_MOESM3_ESM.tif]
